# Doctors and Doctors

**Published:** 1903-11

**Authors:** 


### DOCTORS AND DOCTORS.

"Be Good," "Do It Now." This is the "strange device" stamped on about a thousand little red-wing reminders I sent out lately to my subscribers, that it takes money to get up and mail them every month the livest and liveliest medical journal anywhere. The great majority of them responded promptly, cheerfully and with a pleasant word, for all of which God bless them. My fears are measurably allayed thereby, that the cold, chilling winds of December would nip the toes of Betty and the baby before I could buy them some shoes and stockings. Those fellows are the Old Guard. I never have to call on them twice. But, do you know, "gentle reader," that there are a lot of doctors—happily in the minority—who are so confounded stupid, or pious, or glum, that they have no sense of humor, and can't take a joke? You've got to talk "United States" to them, with the bark on. Some of them get mad at being requested to "do it now," to "be good." They stop their subscription—that is, a few of them take the journal out of the office year after year, and when I send them a bill, about twice a year, they are like brer fox in Uncle Remus' story of the Wonderful Tar Baby, they "keep on ain't sayin' nothin' "; and by and bye I draw a draft on them, and whoopee! how mad some of them do get, to be sure. They make all kinds of claims: "Only subscribed for one year," "didn't get it regular," "bill not correct;" and one fellow, of whom I had a high opinion, wrote on back of draft "I won't pay it." Well, most of these fellows I scratch off and write opposite their name in blue pencil "D. B." Funny thing. "Honesty is not only the best policy, but it pays best," as the fellow said, adding, "I have tried both." "Dan'els," as many call ye editor of the "Red-Back," is often, very often, consulted by insurance companies, manufacturers, mine owners and others—sometimes even by Governors—who have occasion to appoint or employ a doctor or so. There came the general manager of a big insurance company into my office lately and said: "Dan'els, do you know Dr. Bigike, of Bigikeville?" "Yes," I said. "What do you know about him?" "I know that he beat me out of five years' subscription to the 'Red-Back,'" I said, dropping a tear about the size of a biscuit. "That's all I wanted to know," said the insurance man, and walked out.

Then there's another fellow writes: "Please discontinue the 'Red-Back.' Im very fond of it, but I am doing it and myself an injustice in taking it, as I never have time to read it." God pity the man who has not time to read a medical journal—and his patients! As Major Pendleton, the famous Confederate artilleryman, would say before the command "fire," "may the Lord have mercy on them." I wrote this party that he was right; he oughtn't to try to read. A man as busy as he needs all his leisure—if he has any—to rest his brain. I advised him to do it, for fear of a sudden rush of ideas to the brain with fatal results.

The live medical journal is to the practicing doctor what the daily paper is to the business man. A doctor who takes no journals, or, what is the same thing, accepts one when ordered and sent him, but refuses to pay for it, is not worthy of the confidence of any community.

Now, I hate to have to tell these things, but, strange as it may seem, there are doctors in Texas who pass as very respectable, who will repudiate a little subscription bill of two, three, five dollars, after having received the journal all those years; and I have indulged them, unasked, for they simply ignore the bills. Five years to wait for a dollar—a year subscription. There are some who, by reason of poor collection or short crops or other causes, really can't pay. When such a person does me and himself the common justice to say so, nothing gives me more satisfaction than to indulge him. If you can't "do it now," boys, say so, and we will "run the keerds."

---

The Transactions of the State Medical Association of Texas for 1903, just issued from the press of The Von Boeckmann-Jones Co., Austin, is in the nature of a stunner. It lays over anything in the way of society transactions extant. It is a strikingly handsome book of viii+635 pages, printed on heavy white paper, in clear new type, and the copy sent me is bound in black Morocco and gilt, with my name on the cover, a presentation copy. Thanks, Dr. West. It contains a full report of the San Antonio meeting in every detail, excellently reported and ably edited, and is singularly clean of typographical errors. Much credit is reflected on the Association, on its excellent Secretary, Dr. H. A. West, of Galveston, on the printers and binders (the great Austin house of Von Boeckmann-Jones Co.) for it is a "show specimen" of high typographical art. Of the contents I can not now speak. It is chuck full of interesting "papers," some of them being of extraordinary value and importance. This book in every respect is a credit to Texas. Hurrah for Texas, "on general principles."

